# Technique and Circuit for Contactless Readout of Piezoelectric MEMS Resonator Sensors

**DOI:** 10.3390/s20123483

**Published:** 2020-06-19

**Authors:** Marco Baù, Marco Ferrari, Habiba Begum, Abid Ali, Joshua E.-Y. Lee, Vittorio Ferrari

**Affiliations:** 1 Department of Information Engineering, University of Brescia, via Branze 38, I25123 Brescia, Italy; marco.bau@unibs.it (M.B.); marco.ferrari@unibs.it (M.F.); 2 INO-CNR, via Branze 45, I25123 Brescia, Italy; 3 Department of Electrical Engineering, City University of Hong Kong, Kowloon, Hong Kong, China; hbegum2-c@my.cityu.edu.hk (H.B.); abidali2-c@my.cityu.edu.hk (A.A.); josh.lee@cityu.edu.hk (J.E.-Y.L.); 4 State Key Laboratory of Terahertz and Millimeter Waves, City University of Hong Kong, Kowloon, Hong Kong, China

**Keywords:** thin-film piezoelectric-on-silicon resonator, aluminum nitride, time-gated technique, contactless interrogation, piezoelectric MEMS resonator, sensors

## Abstract

A technique and electronic circuit for contactless electromagnetic interrogation of piezoelectric micro-electromechanical system (MEMS) resonator sensors are proposed. The adopted resonator is an aluminum-nitride (AlN) thin-film piezoelectric-on-silicon (TPoS) disk vibrating in radial contour mode at about 6.3 MHz. The MEMS resonator is operated in one-port configuration and it is connected to a spiral coil, forming the sensor unit. A proximate electronic interrogation unit is electromagnetically coupled through a readout coil to the sensor unit. The proposed technique exploits interleaved excitation and detection phases of the MEMS resonator. A tailored electronic circuit manages the periodic switching between the excitation phase, where it generates the excitation signal driving the readout coil, and the detection phase, where it senses the transient decaying response of the resonator by measuring through a high-impedance amplifier the voltage induced back across the readout coil. This approach advantageously ensures that the readout frequency of the MEMS resonator is first order independent of the interrogation distance between the readout and sensor coils. The reported experimental results show successful contactless readout of the MEMS resonator independently from the interrogation distance over a range of 12 mm, and the application as a resonant sensor for ambient temperature and as a resonant acoustic-load sensor to detect and track the deposition and evaporation processes of water microdroplets on the MEMS resonator surface.

## 1. Introduction

Micro-electromechanical system (MEMS) resonators have been intensively investigated in recent years for an increasing number of applications, ranging from timing [1], filtering [2], and actuating to sensing [3]. One advantage of the emerging adoption of MEMS resonators is their potential level of integration with current integrated circuit (IC) technology [4] with respect to other devices, such as surface acoustic wave (SAW) [5,6] or quartz crystal [7] resonators.

Different actuation and detection mechanisms have been adopted for MEMS resonators, such as electrostatic, magnetic, piezoresistive or piezoelectric [8,9]. In particular, piezoelectric materials, such as lead-zirconate-titanate (PZT) films, have been successfully deposited on silicon resonators, either as post-processed thick films [10,11,12], or thin films [13]. In addition, the deposition of other piezoelectric materials, such as zinc oxide (ZnO) or aluminum nitride (AlN) [14], has been demonstrated. In particular, AlN thin-film piezoelectric-on-silicon (TPoS) resonators have attracted great interest because of their better compatibility with IC fabrication processes compared to both PZT and ZnO films [15].

One additional advantage of piezoelectric resonators, especially when operated as sensors, is their suitability for contactless interrogation, i.e., excitation and detection of mechanical resonances without required wired connections [16]. Contactless operation is particularly attractive when cabled connections are unpractical, such as operation in closed environments, sealed packages or in-liquid operation in enclosed volumes. Contactless interrogation exploits the magnetic coupling between a readout coil, which is coupled to the reading circuitry forming the interrogation unit, and a sensor coil, which is connected to the piezoelectric resonator forming the sensor unit. Effective contactless interrogation in real applications demands for the readout of the resonant frequency of the resonator to be independent from the stand-off distance between the interrogation and sensor units. For this purpose, techniques both in the frequency [17] and time [18] domains have been investigated.

Frequency domain techniques typically rely on simultaneous excitation and detection phases with a reflected impedance measured at the readout coil [19,20]. Time domain techniques, also referred to as time-gated, excite the sensor for a finite time duration, and then detect the decaying resonant response, from which the resonant frequency and possibly the quality factor are extracted. Both techniques, besides being applicable also to capacitive sensors [17], have been demonstrated to be to first order independent from the interrogation distance. Additionally, residual dependence on the distance, mainly due to parasitic capacitance in parallel to the readout coil introduced by electrical connections and the front-end electronic circuit, can be mitigated electronically by suitable compensation circuits [21].

In this work the time-gated contactless interrogation technique is innovatively applied to a TPoS contour-mode MEMS resonator based on AlN [22,23]. The resonator is operated in one-port configuration and the proposed technique is verified and experimentally validated through a purposely-designed electronic circuit that is tailored to the electrical parameters of the microresonator. The TPoS resonator is applied as a sensor to the measurement of ambient temperature and the detection of acoustic loading due to microdroplets of deionized (DI) water deposited on the resonator top surface, with real-time tracking of the subsequent evaporation. The proposed system can advantageously operate as a stand-alone device which can be electromagnetically linked for interrogation, without requiring on-board active electronics. The fabrication of the AlN TPoS resonator, the results of finite element simulations, the operating principle of the contactless interrogation technique and electronic circuit, the results of the experimental characterization and the conclusions are reported in Section 2, Section 3, Section 4, Section 5 and Section 6, respectively. Appendix A summarizes the main symbols adopted in the paper.

## 2. Technology and Fabrication Processes

**Figure 1:**
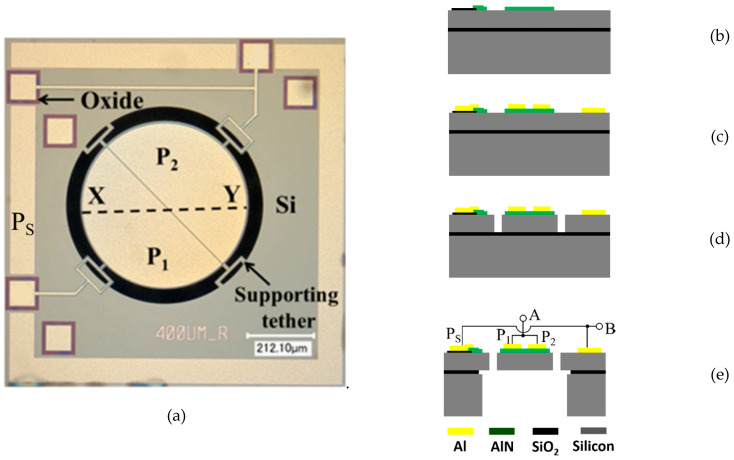
Optical micrograph of the adopted AlN (aluminum nitride) TPoS (thin-film piezoelectric-on-silicon) MEMS (micro-electromechanical system) resonator (**a**). Deposition and patterning of 200 nm thermal oxide followed by deposition and patterning of 500-nm-thick AlN film (**b**). Deposition and patterning of 20 nm +1 µm-thick Cr/Al metal stack (**c**). Front side DRIE (deep reactive ion etching) of 10-µm-thick silicon device layer (**d**). Back side DRIE of silicon substrate followed by buffered HF (hydrogen fluoride) etch of the buried oxide layer to release the device (**e**).

Figure 1a shows the optical micrograph of the adopted MEMS resonator seen from the top. The MEMS resonator is an AlN TPoS disk with a radius of 400 µm, fabricated using the PiezoMUMPs (Piezo Multi-User MEMS Processes) foundry process offered by MEMSCAP [24]. The process begins with a silicon-on-insulator (SOI) wafer and a silicon device layer (Si) that is 10-µm thick. The device layer is a surface-doped n-type layer in order to create an ohmic layer in a subsequent metallization stage to define the contact pads and electrodes. A 200-nm-thick thermal oxide is grown and patterned by reactive ion etching as an insulation layer on the silicon device layer, as depicted in Figure 1b. This is then followed by deposition of AlN as the piezoelectric material by reactive sputtering with a thickness of 500 nm. The AlN layer is patterned by wet etching. The use of AlN has the advantage of having the potential for chip level integration and CMOS compatibility compared to other piezoelectric materials like PZT and ZnO. The 10-µm-thick silicon layer increases energy storage to the resonator compared to a structure that is comprised of only AlN, which generally yields higher quality factors. Next, a metal stack of 20-nm-thick Cr and 1-µm-thick Al is patterned and deposited by a lift-off process to define the contact pads and top electrodes of the resonator, as shown in Figure 1c. Next, the silicon device layer is lithographically patterned and etched by deep reactive ion etching (DRIE) to define the features of the resonator as depicted in Figure 1d. This includes the T-shape tethers on 4 sides of the disk resonator for supporting the structures, as indicated in Figure 1a. The resonator has to be supported from the sides in this case as it is not possible to clamp the structure from the center due to the release method. To release the structure from the substrate, the top side of the wafer is covered with a protective polymer coat first. The back of the SOI wafer is lithographically patterned to define the opening of a cavity, through which the handling layer of the wafer is trench etched by DRIE followed by wet oxide removal of the buried oxide layer. The front side protective coat is then removed to release the device, as shown in Figure 1e, followed by dicing. In this case, the back cavity is circular, which can be viewed in Figure 1a as the dark ring around the TPoS disk. The two Al top electrodes P_1_ and P_2_, and the bottom ground electrode P_S_ allow the operation of the device as either a two-port resonator or, by shorting P_1_ and P_2_, as a one-port resonator between the connections A and B, as shown in Figure 1e. By relating the top view of the device in Figure 1a, with a side view schematic in Figure 1e, it can be seen that the contact pads of P_1_ and P_2_ are isolated from the silicon device layer by 200-nm thermal oxide, while the wide frame tracks around the resonator contact the silicon device layer as grounding pads (P_S_).

## 3. FEM Simulations

**Figure 2:**
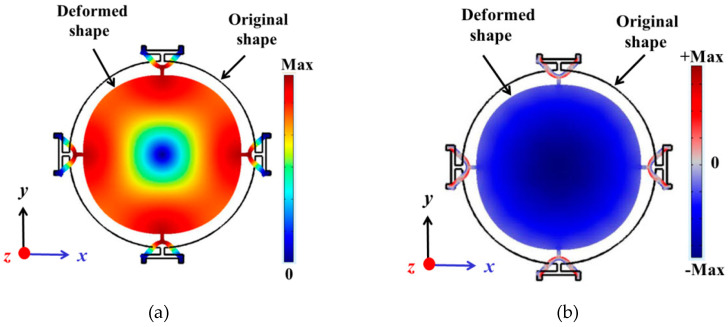
Top view of the round plate with a radius of 400 µm showing the finite element (FE) eigenfrequency simulation of the radial contour mode: total displacement profile associated with the mode (**a**), and corresponding profile of the sum of lateral strains (**b**). The continuous black line outlines the undeformed disk.

The considered device, with the configuration of the electrodes shown in Figure 1b, vibrates in the so-called radial contour mode. The device was investigated by finite element (FE) analysis simulations using COMSOL Multiphysics. An eigenfrequency analysis to compute the mode shape (eigenfunction) and corresponding modal frequency (eigenfrequency) was performed first. Figure 2a shows the total displacement profile of the fundamental radial contour mode, which can be described by the disk contracted and expanding radially with a nodal point at the center. Figure 2b shows the corresponding distribution of the sum of lateral strains, where the maximum strain is concentrated at the center of the disk for the radial contour mode. The resonant frequency scales inversely with the diameter or radius of the disk. The elasticity properties and density of the structure, which determine the resonant frequency, are dominated by the silicon device layer, which is much thicker than the other constituent layers. In the FE model, to compute the resonant frequency, the elastic matrix of single-crystal silicon for the (100) plane, based on the material properties reported in reference [25], has been adopted instead of assuming isotropic elasticity. In the case of the device presented in this paper, which has a disk radius of 400 µm, the resonant frequency of the radial contour mode obtained by FE is 6.32 MHz.

**Figure 3:**
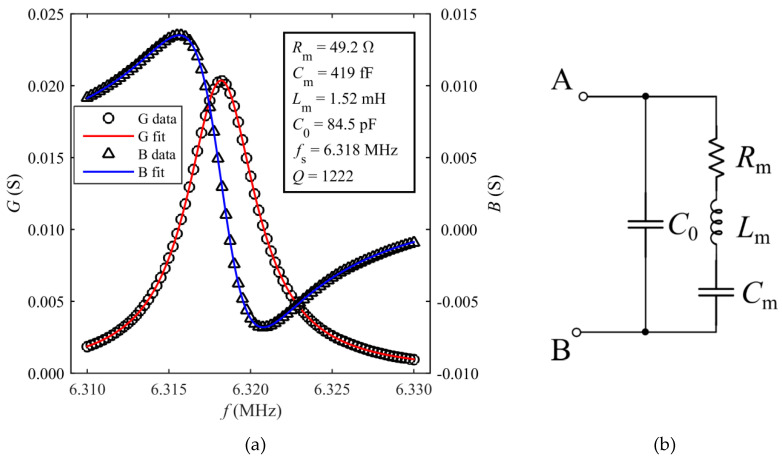
Conductance (*G*) and susceptance (*B*) spectra of TPoS resonator derived from the FE simulations around resonance (**a**). The simulation data have been fitted to theoretical curves and the parameters of the modified Butterworth–van Dyke (BVD) equivalent circuit have been derived. BVD equivalent circuit modeling the TPoS resonator around resonance (**b**).

Subsequently, a frequency domain analysis was performed to compute the electrical admittance *Y = G +* j*B* of the resonator, where *G* and *B* are the conductance and susceptance, respectively. For this purpose, the resonator was driven by a harmonic fixed-frequency voltage across A and B to determine the corresponding value of electrical admittance. By sweeping the driving frequency in the range from 6.310 to 6.330 MHz, the spectra of *G* and *B* were derived as shown in Figure 3a. The electromechanical behavior of the TPoS resonator can be modeled around its resonant frequency through the equivalent Butterworth–van Dyke (BVD) circuit of Figure 3b. The BVD circuit is composed of an electrical branch formed by the capacitance *C*_0_ plus a motional branch in parallel. The motional branch comprises the series inductance *L*_m_, capacitance *C*_m_ and resistance *R*_m_, which represent the equivalent mass, compliance and energy losses of the resonator, respectively [9]. The values of *Y* from the simulation have been fitted with the corresponding expressions of *G* and *B* given by Equations (1) and (2):

(1) $$G=\frac{R_{m}}{R_{m}^{2}+{\left(2\pi fL_{m}-\frac{1}{2\pi fC_{m}}\right)}^{2}},$$

(2) $$\;B=2\pi fC_{0}-\frac{\left(2\pi fL_{m}-\frac{1}{2\pi fC_{m}}\right)}{R_{m}^{2}+{\left(2\pi fL_{m}-\frac{1}{2\pi fC_{m}}\right)}^{2}},\;$$

where *f* is the frequency in hertz. From the fitting, the values of the parameters of the equivalent BVD circuit have been estimated. The resulting fitted curves and the estimated parameters are shown in Figure 3a. From these values, the resonant frequency and the quality factor of the resonator can be calculated as Equations (3) and (4):

(3) $$f_{S}=\frac{1}{2\pi \sqrt{L_{m}C_{m}}},$$

(4) $$\;Q=\frac{1}{R_{m}}\sqrt{\frac{L_{m}}{C_{m}}}.\;$$

Accordingly, the simulated resonant frequency and the quality factor result *f*_S,sim_ = 6.318 MHz and *Q*_sim_ *=* 1222, respectively.

## 4. Operating Principle of the Interrogation Technique and Electronic Circuit

**Figure 4:**
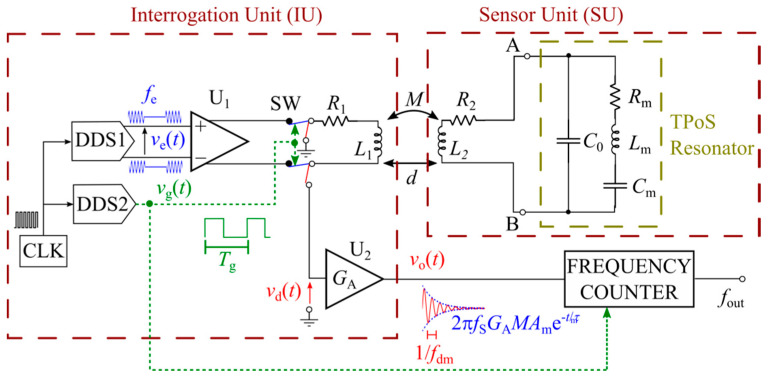
Schematic diagram of the interrogation system and developed electronic circuit.

Figure 4 shows the block diagram of the developed contactless interrogation system, which is composed of an interrogation unit (IU) and a sensor unit (SU). The IU is in turn composed of two programmable direct digital synthesizers (DDSs) named DDS1 and DDS2, fed by the common clock CLK, which generate the relevant signals for system operation. DDS1 generates the sinusoidal waveform *v*_e_(*t*) at frequency *f*_e_, gated by the square waveform *v*_g_(*t*), with period *T*_g_ = *T*_e_ + *T*_d_ generated by DDS2. During the time interval *T*_e_, the interrogation system is in the excitation phase. Consequently, the switch SW is closed and the fully differential amplifier U_1_ drives the readout coil, modeled by the inductance *L*_1_ and series resistance *R*_1_. During the time interval *T*_d_, the interrogation system is in the detection phase. The switch SW is open and the readout signal *v*_d_(*t*) across the readout coil is sensed by the high-impedance amplifier U_2_ and amplified with gain *G*_A_ = 8. The output voltage *v*_o_(*t*) is fed to a frequency counter to obtain the readout frequency *f*_out_. 

The readout coil is electromagnetically air-coupled to the sensor coil modeled by the inductance *L*_2_ and series resistance *R*_2_. The magnetic coupling between *L*_1_ and *L*_2_ is represented by the mutual inductance *M = k(L*_1_*L*_2_)^1/2^, where *k* is the coupling factor. The sensor coil is connected to the resonator represented by its BVD circuit. The sensor coil and the resonator form the SU.

During the excitation phase, through the electromagnetic coupling between *L*_1_ and *L*_2_, the sensor is excited at a frequency *f*_e_ proximal to its series resonant frequency given by Equation (3), i.e., *f*_e_ ≈ *f*_S_. During the detection phase, the excitation signal is disconnected, and the resonator undergoes decaying oscillations at the damped frequency *f*_dm_, forcing a current in *L*_2_. Consequently, an induced readout voltage *v*_d_(*t*) can be sensed across *L*_1_, i.e., at the input of U_2_. It has been demonstrated that the signal *v*_d_(*t*) is the sum of two damped sinusoidal signals at frequency *f*_dm_ and *f*_de_ with exponential decay times *τ*_m_ and *τ*_e_, respectively [18]. The damped sinusoid at *f*_dm_ is due to the mechanical response of the resonator, while the damped sinusoid at *f*_de_ is due to *L*_2_ resonating with *C*_0_. Considering for the resonator the typical values of the BVD model of Figure 3b, for suitable values of *L*_2_ and *R*_2_, it occurs that *τ*_m_ >> *τ*_e_, i.e., the damped sinusoid at *f*_de_ decays rapidly to zero with respect to the damped sinusoid at *f*_dm_. Hence, the contribution of the damped sinusoid at *f*_de_ to *v*_d_(*t*) can be neglected. In addition, assuming infinite input impedance of U_2_, |*R*_2_ + j2π*f*_S_*L*_2_| << 1/(2π*f*_S_*C*_0_) and *R*_m_ << 1/(2π*f*_S_*C*_0_), i.e., at *f*_S_, the impedance of *C*_0_ can be neglected with respect to the corresponding impedance of *R*_2_-*L*_2_, and to that of the motional branch *R*_m_-*L*_m_-*C*_m_ of the BVD circuit, the following simplified expression for *v*_o_(*t*) can be derived [18]:

(5) $$v_{o}\left(t\right)=G_{A}v_{d}\left(t\right)\approx G_{A}\left[2\pi f_{S}MA_{m}e^{-t/\tau_{m}}\cos\left(2\pi f_{\mathrm{dm}}t+\theta_{m}\right)-L_{1}i_{L1}\delta \left(t\right)\right],$$

where the amplitude and phase coefficients *A*_m_ and *θ*_m_ are functions of both the initial conditions taken at the end of the excitation period and the electrical and mechanical parameters of the system composed of *R*_1_, *L*_1_, *R*_2_, *L*_2_ and the resonator. In Equation (5), the damped frequency is *f*_dm_ = [*f*_S_^2^ − 1/(2π*τ*_m_)^2^]^1/2^, with *f*_S_ given by Equation (3), while *τ*_m_ is related to the mechanical quality factor *Q* of the resonator in Equation (4) by *Q* = π*f*_S_*τ*_m_ [16]. It has been demonstrated [18] that if *L*_m_ >> *L*_2_ and for *Q* in the order of 1000, it results *f*_dm_ ≈ *f*_S,_ with (*f*_S_ − *f*_dm_)/*f*_S_ in the order of 10^−7^_._ The additional term *G*_A_$L_{1}i_{L1}\delta \left(t\right)\;$ in Equation (5) is a voltage impulse which accounts for the initial current in *L*_1_. 

It should be noticed that the exact knowledge of the resonant frequency of the resonator is not strictly required to set the excitation frequency *f*_e_ because the measurement technique exploits the free response of the resonator which is independent of the type of excitation. However, if *f*_e_ is close to *f*_S_, the signal-to-noise ratio (SNR) during the detection phase is enhanced, and hence, in principle, a higher interrogation distance *d* can be attained. Equation (5) also shows that *v*_o_(*t*) is proportional to *f*_S_ and the mutual inductance *M* advantageously only acts as a scaling factor on the amplitude of the signal without affecting the value of *f*_dm_. This is a key feature of the proposed technique that makes it possible to derive *f*_dm_ independently from the distance *d*. The frequency *f*_dm_ can be measured through a frequency counter gated with the signal *v*_g_(*t*) to synchronize with the transient and give the readout frequency *f*_out_ = *f*_dm_.

## 5. Results and Discussion

**Figure 5:**
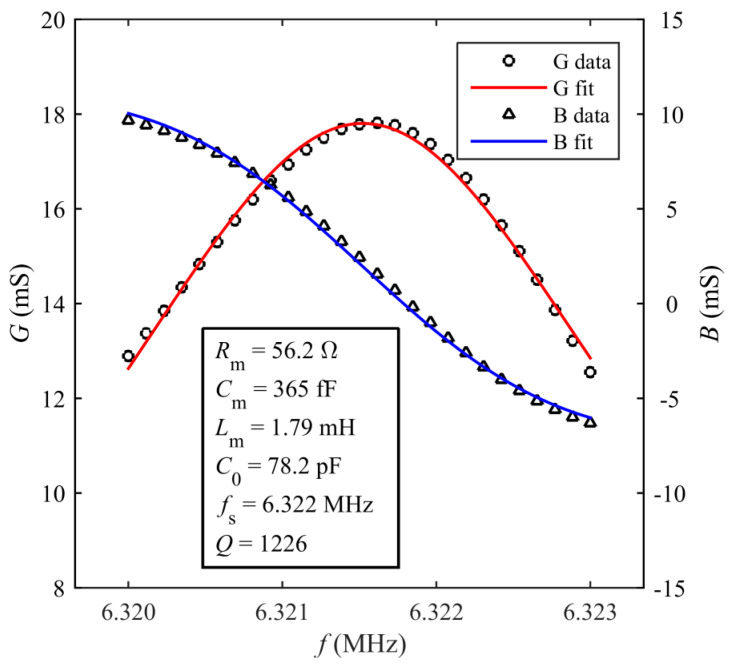
Measured values of the real (*G*) and imaginary (*B*) parts of the admittance of the TPoS resonator around the resonant frequency *f*_S_ of the first vibration mode and corresponding fitted curves. The inset reports the parameters of the BVD model derived from the fitting of the measured data.

Preliminarily, the resonator was electrically characterized by measuring the real and imaginary parts of the admittance *Y* = *G* + j*B* between A and B through contact probes by means of a HP4194A impedance analyzer (Palo Alto, CA, USA). The measurements were taken with the resonator in air at room temperature and atmosphere. The measured spectra of *G* and *B* around the frequency of the first vibration mode are reported in Figure 5, where, consistently with the BVD model, the resonant frequency has been taken in correspondence of the maximum of *G*, i.e., *f*_S_ = 6.322 MHz. The parameters of the BVD equivalent circuit were extracted from the fitting of Y to the measured data, resulting in *R*_m_ = 56.2 Ω, *C*_m_ = 365 fF, *L*_m_ = 1.79 mH, *C*_0_ = 78.2 pF. From these values, a quality factor *Q =* 1226 can be estimated. Compared to the FE simulation results, it can be observed that the experimental values of the parameters of the BVD circuit are in agreement within a few percent. Hence, it also results that *f*_S_ ≈ *f*_S,sim_ and *Q* ≈ *Q*_sim_. 

**Figure 6:**
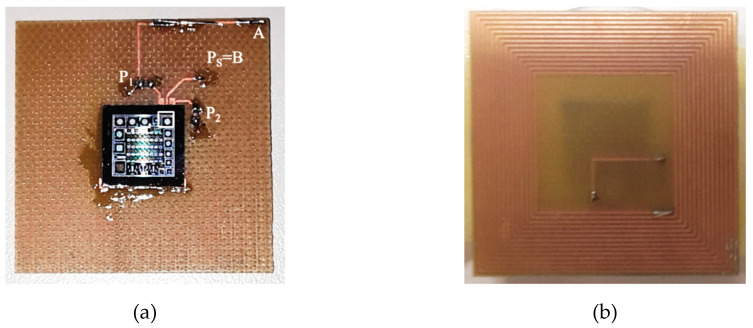
Picture of the fabricated sensor unit: die with the adopted resonator on the top side of the spiral coil (**a**). Bottom side of the spiral coil connected to the resonator (**b**).

Figure 6 shows the fabricated SU. A planar spiral coil has been milled from a copper-clad flame-retardant (FR4) substrate with dimensions of 35 mm × 35 mm. The coil has been electrically characterized at *f*_S_, where it has an equivalent resistance *R*_2_ = 7.23 Ω and inductance *L*_2_ = 8.19 µH. The silicon chip with the TPoS resonator has been glued at the center of the spiral coil and bonded through gold wires to the connection pads.

A prototype of the IU, corresponding to the schematic diagram of Figure 4, was fabricated. In particular, the IU adopts two AD9834 (Analog Devices, Norwood, MA, USA) for the DDSs, fed by a 40 MHz clock. The amplifiers U1 and U2 are an AD8139 (Analog Devices, Norwood, MA, USA) and an OPA656 (Texas Instruments, Dallas, TX, USA), respectively, while the switch SW is a MAX303 (Maxim Integrated, San Jose, CA, USA). The readout coil is a spiral coil on FR4 substrate with *R*_1_ = 5.12 Ω and *L*_1_ = 8.5 µH measured at *f*_S_. The readout frequency *f*_out_ was measured by means of a PM6681 (Fluke, Everett, WA, USA) frequency counter. 

**Figure 7:**
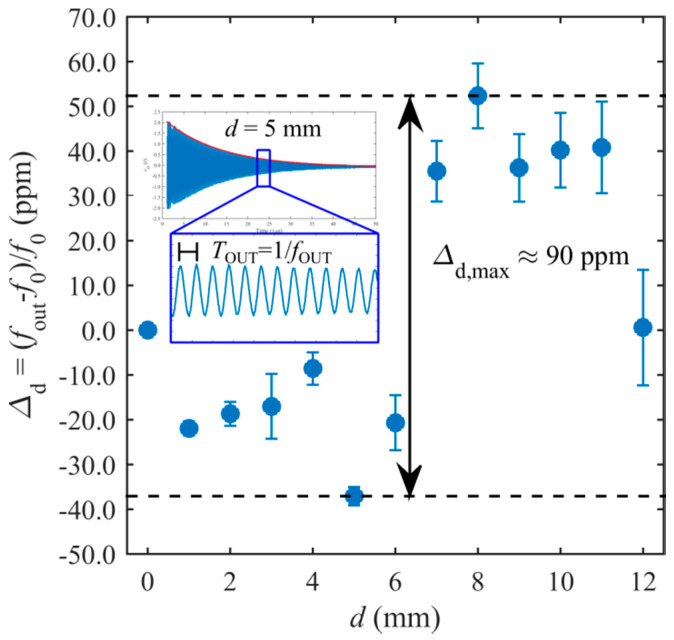
Measured *Δ*_d_ for different values of the interrogation distance *d*. In the inset, the typical waveform of *v*_o_(*t*) is shown.

Initially, the system was tested to verify the independence of *f*_out_ from the interrogation distance *d*. For this purpose, measurements were taken at several prescribed values of *d* in the range between 0 mm and 12 mm. Figure 7 reports the resulting *Δ*_d_ *=* (*f*_out_ – *f*_0_)/*f*_0_, i.e., the measured relative deviation of *f*_out_ with respect to *f*_0_ = 6.355 MHz, where *f*_0_ corresponds to *f*_out_ at *d =* 0 mm. 

For each value of *d*, ten repeated measurements have been acquired and the corresponding standard deviation *σ*(*d*) was calculated. The error bars in Figure 7 represent the relative standard deviation, i.e., *σ*(*d*)/*f*_out_. As it can be observed, in the explored range of distances, the maximum variation of *Δ*_d_ is about *Δ*_d,max_ = 90 ppm, validating the theoretical predictions of the model in Section 2, according to which *f*_out_ does not depend on *M*, and hence, on *d.* The residual difference between *f*_0_ and *f*_S_ can be ascribed to secondary effects not accounted for in the proposed model of the interrogation principle. The inset of Figure 7 shows the typical waveform of *v*_o_(*t*) during the detection phase, corresponding to *d* = 5 mm. As expected, *v*_o_(*t*) is a damped sinusoid at frequency *f*_out_ *= f*_dm_.

To validate the interrogation principle and the capability of the TPoS resonator system to operate as a contactless sensor, two tests have been proposed and performed.

**Figure 8:**
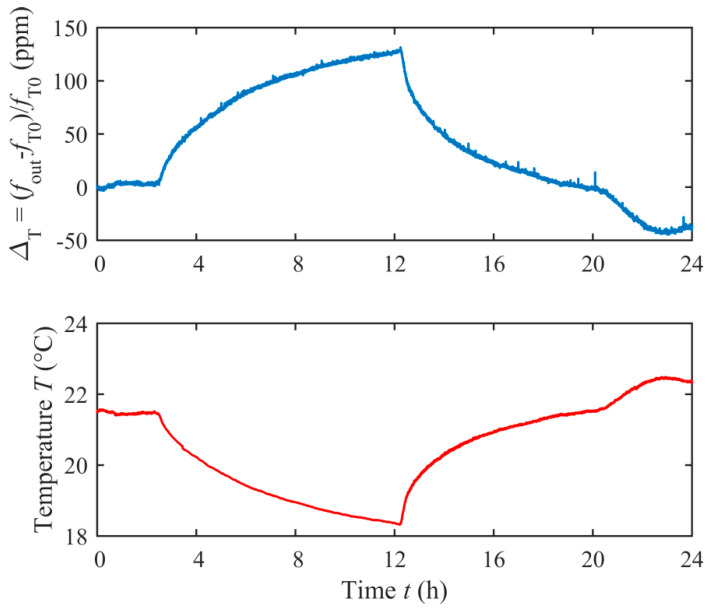
Measured *Δ*_T_ and temperature *T* over a time period of 24 h.

**Figure 9:**
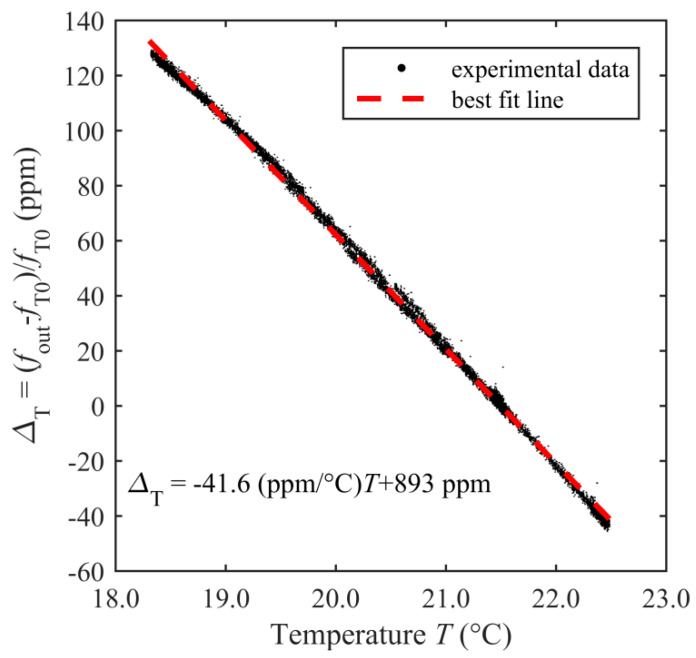
Measured *Δ*_T_ versus temperature *T*.

Firstly, the system was tested for temperature sensing, by acquiring *f*_out_ and the ambient temperature *T* by means of a Pt1000 sensor close to the resonator. Figure 8 shows *Δ*_T_ *=* (*f*_out_ – *f*_T0_)/*f*_T0_, i.e., the relative deviation of *f*_out_ with respect to *f*_T0_, where *f*_T0_ is *f*_out_ at *t* = 0, over a period of 24 h, while the resonator undergoes ambient temperature changes. It can be observed that *Δ*_T_ follows the temperature *T*, with an opposite correlation evidenced by Figure 9, which plots *Δ*_T_ as a function of *T*, similarly to the results in reference [26]. The best fit line of the experimental data allows estimating, for the considered temperature range, a temperature coefficient of frequency (TCF) of −41.6 ppm/°C. 

**Figure 10:**
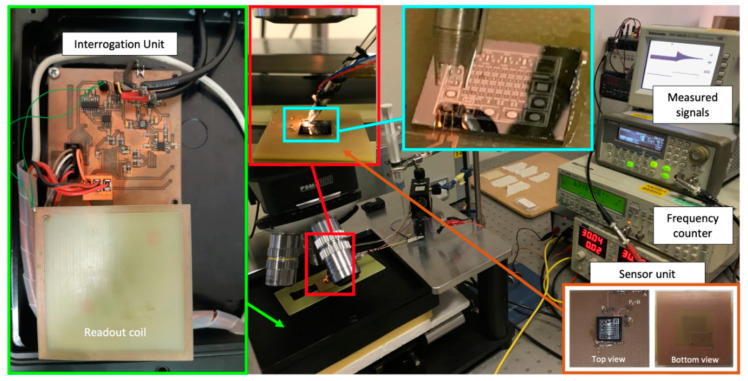
Experimental setup for the deposition of the microdroplets on the top surface of the TPoS resonator.

Subsequently, the system was applied to the measurement of frequency changes due to the deposition of different volumes of DI water microdroplets on the top surface of the TPoS resonator [27]. Figure 10 shows the experimental setup, which adopts a MJ-AB-80 piezoelectric microdispenser (MicroFab Technologies, Inc., Plano, TX, USA) with 80-µm-diameter orifice to deposit 150 pl microdroplets of water on the sensor surface [28]. The microdispenser was driven by a trapezoidal pulse train waveform by means of a programmable function generator and a custom designed power amplifier. Each driving pulse has an amplitude of 60 V and a duration of 25 μs, with rise and fall times of 3 μs, and this causes the ejection of a single droplet. The adopted pulse-train waveform was composed of *N* pulses at the driving frequency of 250 Hz, with *N* that can be set. The SU has been placed under a microscope to align the microdispenser to the resonator surface and take pictures of the microdroplets during the tests. The IU, and hence, the readout coil were kept at *d* = 5 mm beneath the sensor coil for all the deposition tests. 

**Figure 11:**
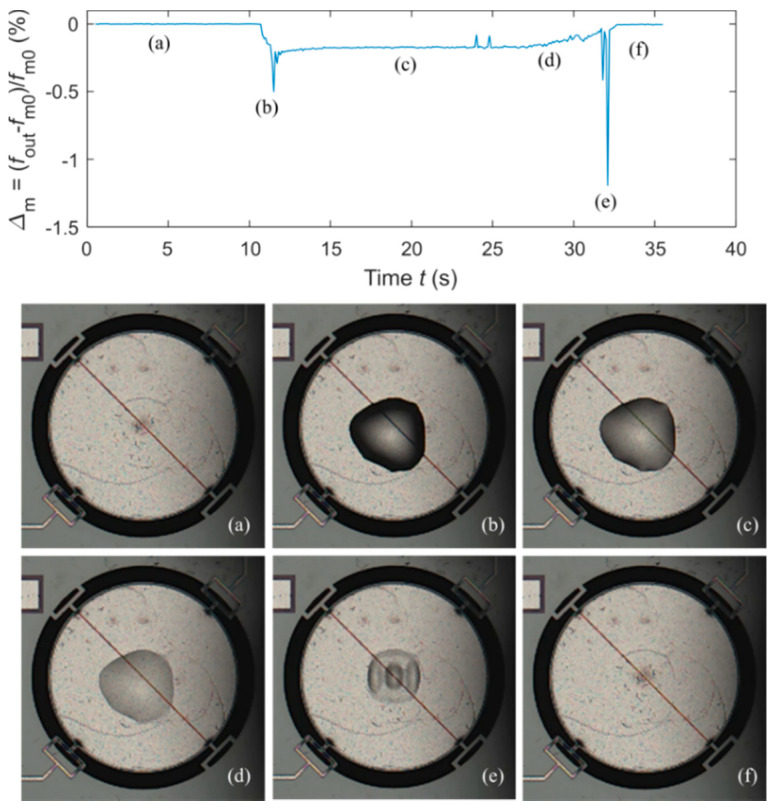
Measured *Δ*_m_ as a function of time for *N =* 100 (upper plot). Pictures taken at the microscope during the deposition and evaporation process of the water microdroplets (**a**)–(**f**).

The upper plot of Figure 11 shows the measured values of *Δ*_m_ *=* (*f*_out_ – *f*_m0_)/*f*_m0_ as a function of time for *N* = 100, i.e., the relative deviation of *f*_out_ with respect to *f*_m0_, where *f*_m0_ is *f*_out_ at *t* = 0, i.e., with no added microdroplets. It can be seen that the response of the resonator, considering also the transient behavior, has a complex pattern. The analysis of such a pattern requires sophisticated modeling [27] which is not considered in the present work that is dedicated to the validation of the time-gated technique innovatively coupled to TPoS resonators. Hence, in the following, only a qualitative discussion of the observed phenomena will be offered. For this purpose, in Figure 11a–f, pictures are shown, taken in correspondence with selected events along the deposition and evaporation process of the droplets.

In particular, the pictures have been respectively taken as listed in the following: (a) at the beginning, when the resonator is dry, hence *Δ*_m_ = 0; (b) after the deposition of the droplets, hence *Δ*_m_ decreases; (c) after a transient, hence *Δ*_m_ settles to a steady state value depending on the amount of the deposited water which, in the meantime, evaporates; (d) when the drying of the surface begins and the water creates a thin film, hence *Δ*_m_ rises; (e) when the drying is almost complete, a fast change in *Δ*_m_ occurs, possibly to be ascribed to the visible change in the spatial distribution of the water film; (f) when the drying is complete, *Δ*_m_ returns to zero. 

**Figure 12:**
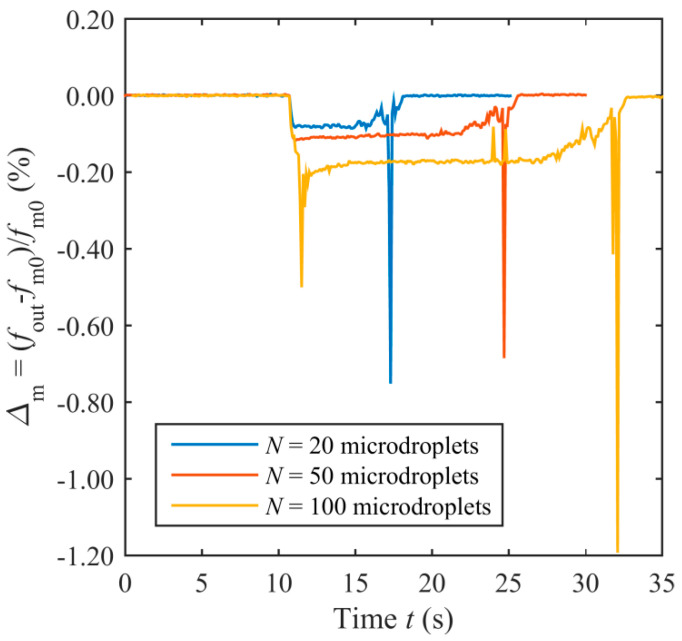
Measured *Δ*_m_ due to different number *N* of microdroplets of DI water deposited on the top surface of the TPoS resonator.

Similar responses have been measured for different volumes of deposited water, as shown in Figure 12, which compares the cases *N =* 20, *N =* 50 and *N =* 100, respectively. As expected, it can be observed that the initial decrease in *Δ*_m_ is related to the volume of deposited water, and hence, to *N*. Similarly, the period of time when *Δ*_m_ has a steady value depends on *N*, being related to the evaporation time. 

It is remarkable that the proposed interrogation technique and system coupled to a TPoS resonator allows the detection of such complex behaviors as was possible with different resonators, namely quartz crystal resonators (QCRs) [29] or resonant piezo-layers (RPLs) [16]. Such an ability is partly due to the inherent speed of data acquisition allowed by the developed technique and electronic system compared to the other techniques, such as for example those based on impedance measurements, which require sampling and computation. In this respect, comparable results have also been obtained by adopting QCRs coupled to oscillator circuits [30,31,32].

In the use of disks resonators as mass-sensitive sensors, it has to be considered that for them, as compared to QCRs and RPLs, due to the involved vibration mode, the mass sensitivity is inherently dependent on both the location and distribution of the added mass on the surface of the resonator. For a distributed mass, the change in frequency depends on the integral of the vibration velocity distribution over the area where the mass is spread [33].

## 6. Conclusions

The reported work has investigated the novel application of the time-gated contactless interrogation technique to a piezoelectric MEMS resonant sensor based on an AlN TPoS resonator operating in the contour-mode and one-port configuration at around 6.3 MHz. The proposed technique exploits the magnetic coupling between two coils respectively connected to a microresonator and an interrogation electronic circuit to perform a gated excitation of the resonator, followed by the sensing of its free decaying response. The adopted MEMS resonator was fabricated in the PiezoMUMPs technology. The resonator was preliminarily investigated by means of FE simulations and subsequently modeled through its BVD equivalent circuit. Simulation results were confirmed by experimental tests on the MEMS device. A dedicated electronic circuit was prototyped by exploiting DDSs to generate both the excitation and gating signals and to manage the interleaved excitation and detection phases. The independence of the readout frequency from the operating distance between the interrogation unit and the sensor unit was experimentally validated over an operating range of 12 mm. The proposed technique and device were successfully demonstrated as a resonant sensor to track the variations of the readout frequency against ambient temperature and as a resonant acoustic-load sensor to detect and record the deposition and evaporation processes of water microdroplets on the MEMS resonator surface.

## Appendix A

**Table A1:** List of the main symbols used in the manuscript.

Symbol	Definition
***f*** **_S_**	Mechanical resonant frequency of the TPoS resonator
***Q***	Quality factor of the TPoS resonator
***f*** **_S,sim_**	Simulated mechanical resonant frequency of the TPoS resonator
***Q*** **_sim_**	Simulated quality factor of the TPoS resonator
***d***	Distance between the readout coil and sensor coil
***f*** **_e_**	Frequency of the excitation voltage *v*_e_(*t*)
***f*** **_de_**	Frequency of the damped sinusoid due to *L*_2_ resonating with *C*_0_.
***f*** **_dm_**	Frequency of the damped sinusoid due to the mechanical response of the resonator
***τ*** **_e_**	Exponential decay time of the damped sinusoid at *f*_de_
***τ*** **_m_**	Exponential decay time of the damped sinusoid at *f*_dm_
***f*** **_out_**	Output frequency measured through the frequency counter
***f*** **_0_**	*f*_out_ taken at *d* = 0 mm
***f*** **_T0_**	*f*_out_ at *t* = 0 s (temperature sensing test)
***f*** **_m0_**	*f*_out_ at *t* = 0 s (microdroplet deposition test)
***Δ*** **_d_**	Relative deviation of *f*_out_ with respect to *f*_0,_ i.e., *Δ*_d_ *=* (*f*_out_ – *f*_0_)/*f*_0_
***Δ*** **_T_**	Relative deviation of *f*_out_ with respect to *f*_T0,_ i.e., *Δ*_T_ *=* (*f*_out_ – *f*_T0_)/*f*_T0_
***Δ*** **_m_**	Relative deviation of *f*_out_ with respect to *f*_m0,_ i.e., *Δ*_m_ *=* (*f*_out_ – *f*_m0_)/*f*_m0_
